# How Patients With Chronic Liver Diseases Succeed to Deal With COVID-19?

**DOI:** 10.3389/fmed.2020.00398

**Published:** 2020-07-10

**Authors:** Sama Rezasoltani, Behzad Hatami, Abbas Yadegar, Hamid Asadzadeh Aghdaei, Mohammad Reza Zali

**Affiliations:** ^1^Foodborne and Waterborne Diseases Research Center, Research Institute for Gastroenterology and Liver Diseases, Shahid Beheshti University of Medical Sciences, Tehran, Iran; ^2^Gastroenterology and Liver Diseases Research Center, Research Institute for Gastroenterology and Liver Diseases, Shahid Beheshti University of Medical Sciences, Tehran, Iran; ^3^Basic and Molecular Epidemiology of Gastrointestinal Disorders Research Center, Research Institute for Gastroenterology and Liver Diseases, Shahid Beheshti University of Medical Sciences, Tehran, Iran

**Keywords:** coronaviruses, COVID-19, infectious disease, liver injuries, chronic liver diseases, medical therapy

## Abstract

The human pathogenic coronaviruses cause infections of the respiratory tract from mild to severe ranges. Mild cases may look like the common cold, while cases with severe disease may represent severe acute respiratory syndrome (SARS), Middle East respiratory syndrome (MERS) and coronavirus disease 2019 (COVID-19). Currently, COVID-19 is a rapidly emerging infection and the number of COVID-19 cases and its associated deaths are quickly growing around the world. COVID-19 infection can involve multiple body organs other than respiratory tract and lungs such as liver. It is hypothesized that COVID-19-associated liver injury can hamper the host drug metabolism and excretion. Liver involvement present with the elevation of enzymatic levels of alanine transaminase (ALT), aspartate transaminase (AST), alkaline phosphatase (ALP), and gamma-glutamyl transferase (GGT) accompanied by enhanced total bilirubin and decreased albumin levels has been reported in COVID-19 cases. One of the major concerns during COVID-19 outbreak is the population with a history of pre-existing liver disorders including viral hepatitis, alcoholic liver disease (ALD), non-alcoholic fatty liver disease (NAFLD), autoimmune hepatitis, hepatic compensated, and decompensated cirrhosis. Herein, we discussed the probable correlation between COVID-19 infection and liver damages, particularly chronic and pre-existing liver diseases during COVID-19 outbreak. Furthermore, we explained about the liver transplant recipients and post-transplant drugs used in patients with COVID-19 infection. Finally, we discussed about the therapeutic medications administered in COVID-19 patients with underlying liver injuries and their significant considerations.

## Introduction

The pathogenic coronaviruses are a class of enveloped, positive-sense, single-stranded RNA viruses, which infect amphibians, birds, and mammals. Coronaviruses in humans result in infections of the respiratory tract from mild to more severe ranges. Mild disease presents like a common cold, while more serious forms can lead to severe acute respiratory syndrome (SARS), Middle East respiratory syndrome (MERS), and coronavirus disease 2019 (COVID-19). Until now, there are no antiviral medications and vaccines to treat or prevent coronavirus associated infections (1–3).

### Structural Characteristics of Coronavirus

**Figure 1:**
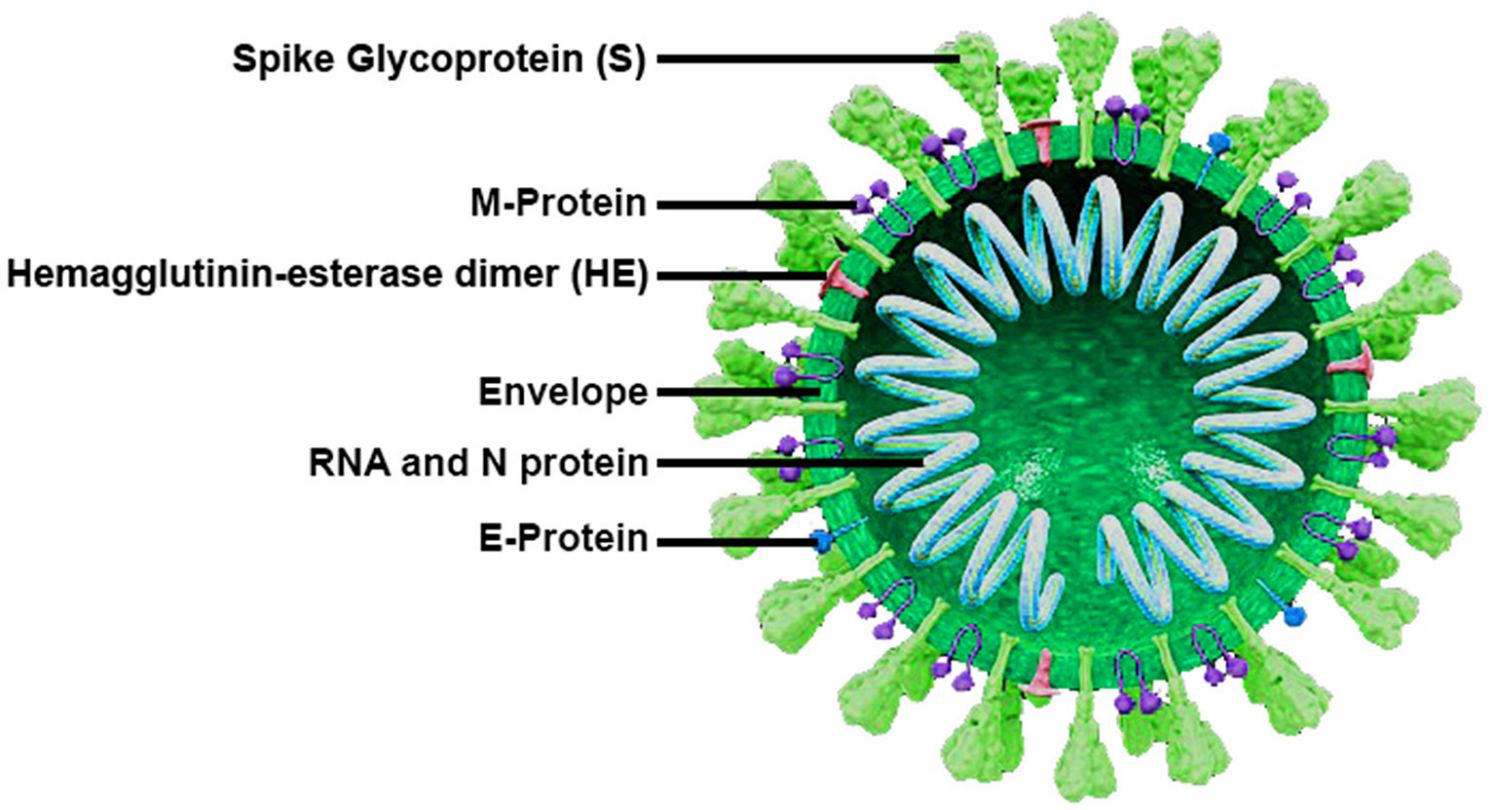
Structural model and the major proteins encoded by the genome of SARS-CoV-2.

Coronaviruses are well-known as large pleomorphic spherical viruses, ~120–140 nm in size (4). The average diameter of the envelope is ~80 nm and the spikes are ~20 nm long (5, 6). The envelope structure is composed of a lipid bilayer, the membrane protein, envelope protein, and the anchored spike surface proteins (7). Also, subgroup A of beta-coronaviruses consists of the members with shorter spike-like structural component named hemagglutinin esterase (HE) (8). All of these components are required to produce a structurally complete viral particle. The coronavirus nucleocapsid is a structural protein which forms complexes with positive-sense single-stranded genomic RNA (Figure 1) (6, 9).

**Figure 2:**
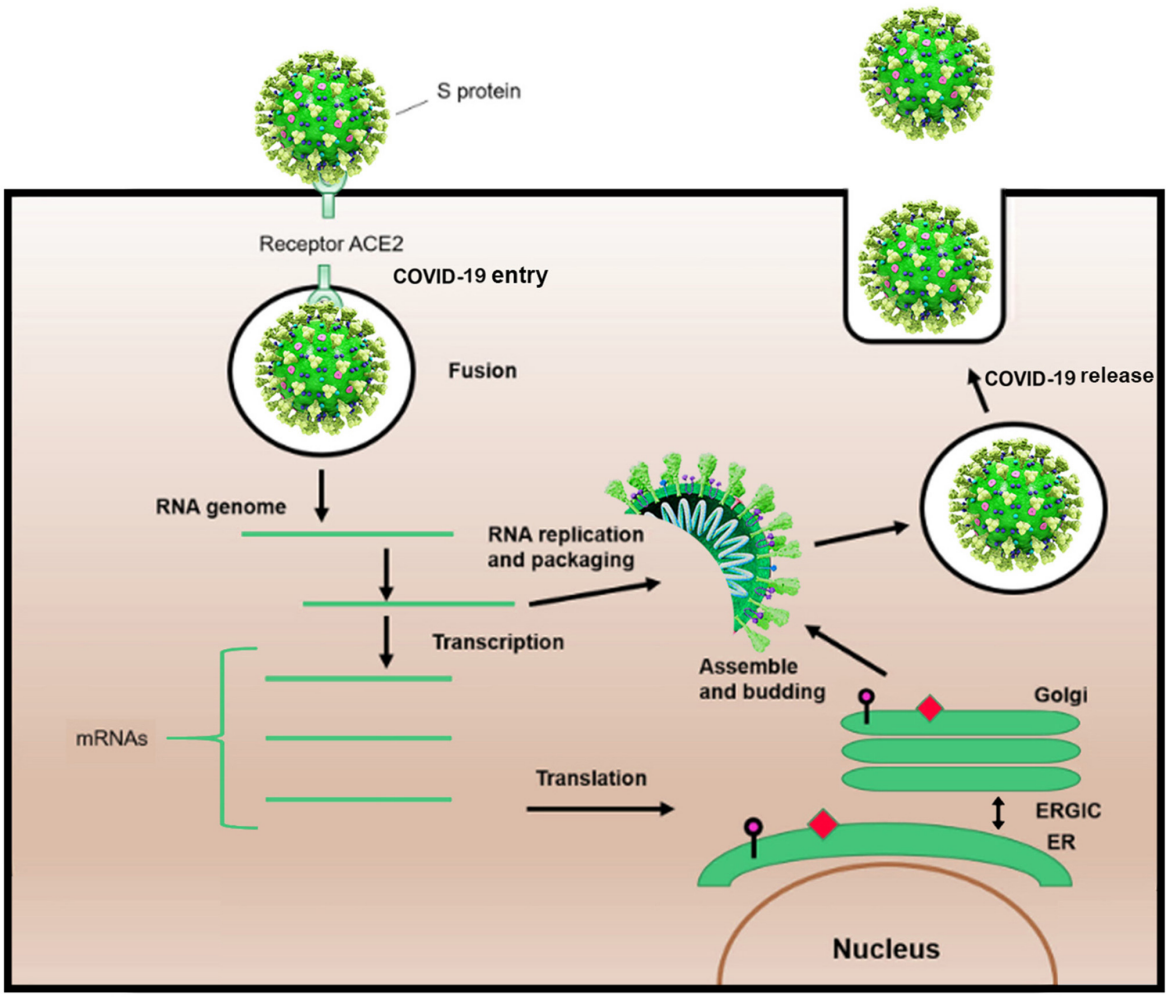
Schematic life cycle of COVID-19. S protein binds to the angiotensin-converting enzyme (ACE2) receptor to simplify the entrance. After the viral fusion, the RNA of the virus is replicated and transcribed into mRNAs. Then, several proteins of the virus are synthesized. Subsequently, viral proteins and RNA genome are assembled in the endoplasmic reticulum (ER) and Golgi apparatus, followed by budding into the lumen of the endoplasmic reticulum-Golgi intermediate compartment (ERGIC). Assembled viruses are ready to release from the infected cells.

Shape analysis of coronavirus proposed that receptor binding domain was contained of an external subdomain and a core (10). The receptor protein, angiotensin-converting enzyme 2, or ACE2, is reported to act as functional receptor for SARS coronavirus 2 (SARS-CoV-2) (11–13). Similar to SARS-CoV, ACE2 is exploited by SARS-CoV-2 as a cellular entry receptor, therefore, inhibition of virues-ACE2 interaction may intercept viral entry into host cells and subsequently prevent COVID-19 infection (Figure 2) (14).

### COVID-19

As mentioned above, the truly dreadful and pathogenic coronaviruses for humans include SARS-CoV, MERS-CoV, and now SARS-CoV-2. The genome sequence of SARS-CoV-2 shares 82% homology to SARS-CoV, and 50% to MERS-CoV (15). These pathogenic coronaviruses result in respiratory, hepatic, neuronal, and intestinal diseases and can cause multiple organ failures, acute respiratory distress syndrome (ARDS) and decease (16). Presently, COVID-19 is a rapidly emerging infection and the number of COVID-19 cases and its associated deaths is quickly rising throughout the world (17). Patients with mild COVID-19 infection represent dry cough, fever, fatigue, diarrhea, and vomiting. In more severe patients, hypoxemia and respiratory distress usually arise a week after onset of illness, and may deteriorate into ARDS, septic shock, metabolic acidosis, and decease (16, 18).

Majority of COVID-19 infected patients have a mild disease, while 14 and 5% of the patients experience serious and critical course, respectively (1). This infection has been reported to be more frequent in males showing a mild course of the disease (19, 20). It has been observed that in the early stage of disease, not remarkable laboratory findings, and clinical symptoms were reported, whereas with the progression of COVID-19 infection, a significant increase in the level of creatine kinase (CK), C-reactive protein (CRP), erythrocyte sedimentation rate (ESR), aspartate transaminase (AST), alanine transaminase (ALT), and lactate dehydrogenase (LDH) has been indicated. Moreover, significant elevation in the count of white blood cells (WBC), serum neutrophil, and decreased hemoglobin, lymphocyte, and eosinophil count could be noted (21).

Several studies have highlighted the lungs as the primary and critical body organ to be affected by SARS-CoV-2. However, as our knowledge on the pathogenesis of SARS-CoV-2 infection continues to rapidly evolve, very limited data exists about the engagement of other body organs and tissues such as liver by COVID-19 infection (17).

## COVID-19 AND Liver

Today, chronic liver diseases are regarded as a major public health concern worldwide. According to the global burden of liver diseases, how various underlying liver conditions affect hepatic damages in COVID-19 cases demands to be seriously investigated (18). Currently, there are insufficient findings about the incidence of chronic liver disease in individuals with COVID-19. In a recent meta-analysis by Mantovani et al. (22), 11 observational studies involving a total of 2,034 cases with COVID-19 were included to evaluate the correlation between liver injury and COVID-19 infection. Overall, the prevalence of chronic liver disease was reported to be 3% (95% CI 2–4%; *I*^2^ = 29.1%) at baseline which was relatively low. Also, patients with more severe disease, patients referred to the intensive care unit (ICU) and patients with ARDS had higher levels of AST and ALT than those with milder disease. For example patients admitted to ICU had higher serum levels of AST (52 IU/L) and ALT (35 IU/L) than those not referred to ICU [i.e., AST: (29 IU/L); ALT (23 IU/L)]. Similarly, the same results were obtained for total bilirubin levels (23).

The incidence of hepatic damage during COVID-19 infection ranged from 14.8% up to 53%, mostly presented by abnormal levels of ALT, AST, alkaline phosphatase (ALP), gamma-glutamyl transferase (GGT) accompanied by increased total bilirubin ranges (24–27). In another retrospective study of 148 consecutive COVID-19 cases, the incidence rates of tests involved in liver function elevation included ALT (18.2%), AST (21.6%), LDH (35.1%), GGT (17.6%), ALP (4.1%), and total bilirubin (6.1%) (24). The level of serum albumin decreased in more severe cases and the range for albumin testing was ~26.3–30.9 g/L (26). In clinical practice, it is necessary to differentiate the onset of abnormal hepatic function, although it occurs at the diagnosis time or in the course of treatment. In addition, lymphopenia, thrombocytopenia, anemia, and coagulation abnormalities as well as low albumin are issues observed in both liver diseases and COVID-19 infection (28).

Hypoalbuminemia has been reported in severe COVID-19 patients. Previously, hypoalbuminemia was considered to be a negative prognostic marker, both in patients with chronic liver disease and in individuals with SARS and MERS. Supplementing of amino acids to a patient's diet is an important step to hepatic albumin synthesis in clinical condition. This highlights the critical roles of nutritional supplements during early stages of COVID-19. Also, the hepatocyte albumin synthesis is downregulated by interaction of cytokines which are produced during cytokine storm induced by SARS-CoV-2. Both mechanisms contribute to intense hypoalbuminemia that, combined with fluid losses because of fever, is responsible for shock and hypovolemia identified in COVID-19 cases in critical care settings (29).

Interestingly, severe COVID-19 patients have higher rates of hepatic disorders (24). The ratio of increasing hepatic damage in severe cases of COVID-19 was remarkably higher than cases with mild disease (18, 30). In deceased COVID-19 cases, the occurrence of liver damage reaches up to 78% (24). In a study from Iran, it was shown that the count of platelets and lymphocytes were notably lower in COVID-19 cases than healthy controls. Also, the levels of ALT, AST, and ALP were higher in COVID-19 patients as compared to healthy group. Moreover, the risk of transfer to ICU and critical care unit (CCU) was forcefully related to increased AST and total bilirubin levels, and the rate of mortality remarkably increased with elevated AST levels (31). It was reported that the elevation of AST level was 62% in ICU cases in contrast to 25% of cases who did not require ICU (18). Furthermore, Shi et al. (32) reported that COVID-19 patients who were diagnosed at subclinical period and before onset of clinical symptoms had considerably lower rate of elevated AST than cases diagnosed after symptom onset. Hence, hepatic damage has been more prevalent in severe COVID-19 patients compared to mild cases (16).

Till now, our knowledge about the pathogenic role of SARS-CoV-2 in hepatic damage is very limited. As aforementioned, similar to SARS-CoV, SARS-CoV-2 exploits ACE2 as its entry receptor (33, 34). Despite high expression of ACE2 receptor on biliary cells, hepatocellular liver injury has been observed in a relatively broad range of patients with COVID-19 (20).

Findings of hepatic damage in COVID-19 patients remain to be seriously investigated. Various mechanisms have been proposed for liver injury during COVID-19 infection; some of them are as follows: (1) immune-mediated hepatitis, (2) direct cytopathic effect (CPE) of the virus, (3) drug-induced liver injury secondary to medications used for the treatment of COVID-19 disease, (4) secondary to infection-induced systemic inflammation, (5) hepatic congestion secondary to positive end-expiratory pressure (PEEP) ventilation, and (6) pre-existing liver injury (15, 35, 36).

Immune dysfunctions including decrease in CD4^+^ T-cell levels; lymphopenia and cytokine storm are prevalent features in COVID-19 patients and may be considered as important factors associated to severity of disease and death (15). Liver damage in severe forms of the disease is associated with activation of coagulation and fibrinolytic cascades, decreased platelet counts, and neutrophil to lymphocyte ratio, as well as increased levels of serum ferritin; although, these biomarkers are all non-specific (4). In severe COVID-19 infection, the plasma levels of CK, LDH, or myoglobin could also be increased. Hence, merely aminotransferase elevations (especially if AST>ALT) in COVID-19 infected patients might have non-hepatic origin such as COVID-19-induced myositis (4).

## COVID-19 and Chronic Liver Diseases

The risk of getting COVID-19 infection in patients with chronic liver diseases and cirrhosis are not well-understood, because such cases have impaired immunity and worse outcomes in ARDS than other of the critically infected and sick population (35, 37). Yet, there has been no report regarding liver injury happening in COVID-19 cases with chronic liver diseases, including chronic hepatitis B virus (HBV) and hepatitis C virus (HCV) (16). This means that for the chronic HBV and HCV patients with viral suppression who are following long-term therapy or are in immunotolerant phases, evidence of hepatic damage, and viral replication after co-infection with SARS-CoV-2 should to be deeply investigated. In addition, effects of glucocorticoids on prognosis of the disease among COVID-19 cases with autoimmune hepatitis need to be evaluated. Also, COVID-19 cases with hepatic cirrhosis or hepatocellular carcinoma (HCC) may be more prone to COVID-19 infection due to their systemic immunocompromised conditions. Severe complications including variceal hemorrhage, hepatic encephalopathy, serious infections, and death rate in such cases demand to be checked in large clinical scales. In view of systemic immunocompromised state, more intensive surveillance and systematic therapeutic approach are required for severe cases of patients with COVID-19 infection with underlying advanced hepatic disease, particularly in elderly cases with comorbidities. Also, subsequent studies should concentrate on the causes of hepatic damage in COVID-19 cases and the impact of concurrent hepatic diseases on therapeutic outcomes of COVID-19 infection (15). Below, we explained more about different types of chronic liver diseases including viral hepatitis, alcoholic liver disease (ALD), non-alcoholic fatty liver disease (NAFLD), autoimmune hepatitis, and liver cirrhosis during COVID-19 outbreak. We also focused on liver transplant recipients and post-transplant drugs used in patients with COVID-19 infection. Finally, we discussed about the therapeutic medications administered in COVID-19 patients with underlying liver injuries and their significant considerations.

### Viral Hepatitis

The European Association for the Study of the Liver (EASL) believes that chronic viral hepatitis does not augment the risk of more severe COVID-19 infection (28); while Xiaoping et al. (19) in a study on 15 HBV patients of 123 COVID-19 cases found that COVID-19-HBV coinfection were more likely to induce hepatic injury with severe consequences and death. The American Association for the Study of Liver Diseases (AASLD) recommends that hepatitis B and hepatitis C patients who are on the antiviral therapy should continue their treatments. Also, treatment for chronic HBV and HCV infection in patients negative for COVID-19 can be offered if clinically indicated. Hepatitis B medication therapy in COVID-19 cases should not be initiated except for a clinical suspicion of hepatitis B flare (38). Interferons (IFNs) have already been administrated for the treatment of MERS and SARS. Recently, application of interferon alfa-2b was investigated against SARS-CoV-2 in its nebulized form among 77 patients (39). Chinese guidelines suggested interferon alfa by nebulization (39). In a recent case series, three patients with severe form of COVID-19 pneumonia also were treated with pegylated interferon alfa-2a (180 μg per week and only 1 or 2 doses subcutaneously) (40). Cases experienced clinical improvement without development to ARDS and more rapid viral clearance. But, it is hard to analogize this finding to broader cases (40). Also, tenofovir which is known as one of the nucleotide reverse transcriptase inhibitors (NRTIs), is extensively used for the treatment of chronic HBV and can bind to SARS-CoV-2 RNA-dependent RNA polymerase (RdRp) with binding capabilities similar to those of native nucleotides. However, its effectiveness as a potential medication against COVID-19 should be thoroughly investigated. In addition, RNA synthesis nucleos(t)ide analog inhibitors, acting as chain terminators of viral RNA, like tenofovir disoproxil fumarate (TDF), abacavir, or lamivudin, potentially could be effective for COVID-19 infection (41). Above all, commencement of direct acting antivirals (DAAs) for chronic HCV in COVID-19 cases is not permitted and it is reasonable to be delayed (42).

### ALD

Although for the time being no specific guideline exists for management of alcoholic hepatitis during the COVID-19 pandemic, caution is recommended in patients taking glucocorticoids therapy in the COVID-19 era. The immunosuppressive effects of corticosteroid therapy for alcoholic hepatitis, also alcohol use disorder (AUD) itself, are regarded as risk factors for more severe disease. The COVID-19 pandemic has also augmented considerable barriers for ALD patients being considered for liver transplantation, resulting in not only problems in maintaining linkage to care but also increased risk for relapse. Regrettably, people who drank alcohol in a misguided effort to protect themselves against COVID-19, were exposed at increased risk of alcoholic hepatitis and raised hundreds of death from acute methanol poising (43). AASLD-COVID-19 Hangover announced patients with ALD may be amongst the affected populations, and being at a higher risk of severe COVID-19 due to having high-risk underlying comorbidities and a potentially depressed immune system. However, it is worthy to be remarked that social isolation and distancing may lead to psychological depression and increased drinking or relapse (44).

### NAFLD

Patients with NAFLD/non-alcoholic steatohepatitis (NASH) often have components of metabolic syndrome such as hypertension, obesity, and diabetes mellitus that put them at higher risk of severe COVID-19 disease (28). It has been demonstrated that COVID-19 patients with NAFLD were at increased risk of COVID-19 disease progression, abnormal liver function, and longer period of viral shedding in comparison with non-NAFLD individuals (19).

### Autoimmune Hepatitis

Patients with autoimmune hepatitis on immunosuppressive agents (prednisone and/or azathioprine) should be considered as a high-risk group for severe COVID-19 infection (38). In these patients, reduction of immunosuppressive therapy is not recommended unless there is a definite indication for it, and the final decision should be taken by a specialist (19, 28, 38). In suspected patients of autoimmune hepatitis based on clinical features and autoantibody profiles, treatment can be offered without histological confirmation (28).

### Liver Cirrhosis

Cirrhotic patients are more suspected to be affected with COVID-19 due to having potentially a hampered immune system than healthy subjects. Cirrhotic patients may develop acute on chronic liver failure (ACLF) due to massive inflammatory responses (45). At the moment, there is very limited data about the incidence of complications in COVID-19 individuals, consisting of upper gastrointestinal (GI) bleeding, liver failure, and hepatic encephalopathy (42, 46). In a study by Xiao et al. (47) performed on decompensated cirrhotic patients in China, none of them had COVID-19 clinical symptoms, when precautionary approaches were taken, specifically protective measures for outpatients, health worker training, new diagnostic procedures, treatment, and emergency strategies. On the other hand, 101 decompensated cirrhotic patients at other hospitals where preventing actions had not been executed announced an incidence of 16.8% of COVID-19 cases. In another study, 81 pneumonia patients containing seven patients with liver cirrhosis, were evaluated by serial chest CT scans and clinical laboratory tests (32). Liver tests containing prothrombin time, serum albumin, platelet count, bilirubin, and fibrinogen did not differ between individuals with and without cirrhosis, but it is required to perform further studies to clarify the effect of cirrhosis on COVID-19 infection (32).

There are limited information about COVID-19 infection in liver cirrhosis; however it is anticipated to be a risk factor for the severe form of COVID-19. Therefore, taking rigid protective actions to prevent SARS-CoV-2 infection in patients with cirrhotic complications is extremely important.

### Compensated Cirrhosis

These patients are expected to be at higher risk for severe COVID-19 infection (38). In order to limit exposure to medical staff, use of telemedicine or phone visits is recommended (28, 38). It is suggested that these patients do not travel during the COVID-19 outbreak (28). In patients with compensated cirrhosis, it is reasonable to defer liver cancer surveillance (by ultrasonography) and screening for varices (by upper endoscopy). Non-invasive risk evaluation of esophageal varices can be a proper alternative (28, 38). Moreover, vaccination for *Streptococcus pneumoniae* and influenza virus is of great importance (28).

## Decompensated Cirrhosis

Decompensated cirrhotic patients are at increased risk for acquiring severe COVID-19 disease as well (28, 38). Standard care according to guidelines is necessary but using telemedicine/phone visits, if possible, can help limit exposure to medical staff (28, 38). Traveling during the COVID-19 pandemic is not recommended (38). Additionally, vaccination for *S. pneumoniae* and influenza should also be emphasized (38). COVID-19 testing for patients with acute decompensation and/or ACLF is indicated (38).

Variceal screening by upper endoscopy in patients without COVID-19 should be restricted to high-risk ones for variceal hemorrhage including cases with a history of variceal bleeding or evidence of clinically significant portal hypertension (ascites, platelet count <100,000/μl and etc.). Otherwise, non-invasive procedures for the prediction of varices can be used (28, 38). In order to reduce the risk of catching and spreading the SARS-CoV-2 infection, endoscopic procedure in COVID-19 patients should be confined to emergencies like GI bleeding or some other serious indications (28). HCC surveillance by ultrasonography should be postponed in cases without COVID-19 infection. However, critical circumstances like elevated levels of alpha-feto-protein (AFP), advanced cirrhosis, chronic HBV, NASH, and diabetes are on the top priority for screening. On the other hand, liver cancer surveillance should be postponed for COVID-19 patients until after improvement (28). Listing for liver transplantation should be confined to ACLF, high model for end-stage liver disease (MELD) scores and HCC at the upper limit of the Milan criteria (28).

## Liver Transplants

Liver transplant recipients are significantly at higher risk for COVID-19 infection. These individuals that are on immunosuppressive drugs are considered to be at higher risk of getting this infection and can terminate with severe disease. On the other hand, transplant recipients may not exhibit symptoms; breathlessness and fever to begin with. Apart from general precautions, they should try to avoid non-essential travel and crowds (48).

Data obtained from Transplant centers revealed that liver transplant patients may experience a lower grade of inflammation and less severe lung injury due to COVID-19 than in non-transplant patients. It is suggested that use of immunosuppressive medications in these patients can modulate the host immune response against viral infection. (49). With respect to liver transplant recipients, potential adverse events of these drugs have to be considered as well. For example, drug monitoring should be performed for blood levels of tacrolimus, cyclosporine, sirolimus, and everolimus in patients taking immunosuppressive treatments (28). Initiation of early treatment may also be a vital step to prevent severe pneumonia in liver transplant patients. In cases with liver disease, it is advised to rapidly get into early antiviral treatment programs. Certain considerations and medications that have been recommended for the treatment of COVID-19 after liver transplantation consist of remdesivir, chloroquine/hydroxychloroquine with or without azithromycin, lopinavir/ritonavir, tocilizumab, methylprednisolone, anakinra and convalescent plasma, favipiravir/favilavir, sofosbuvir with/without ribavirin, baricitinib, camostat, emapalumab, and anakinra based on EASL-ESCMID reports (28).

## Medical Treatment in COVID-19 Patients With Liver Damages

Similar to MERS and SARS, antivirals, steroids, and antibiotics are taken for the treatment of COVID-19 infection. Such medications are possible causes of hepatic damage during COVID-19 disease, though this needs further investigations (27). Until now, there is no well-established therapy for COVID-19 infection, and the present therapeutic regimens offered for COVID-19 cases are the ones which have formerly been successful in SARS and MERS. Currently, drugs that are widely suggested for the treatment of COVID-19 infection include chloroquine/hydroxy chloroquine with or without azithromycin, lopinavir/ritonavir, ribavirin, favipiravir, remdesivir, and monoclonal antibodies such a tocilizumab (28). Most of these medicines are metabolized in the liver. Hence, liver injury can enhance the risk of drug toxicity in these patients. It should be noted that patients with chronic liver disease, particularly Child-Pugh B/C cirrhosis, are more likely prone to adverse reactions of over mentioned medications (50). Hence, precise and repeated monitoring of liver biochemistries in COVID-19 patients can result in early diagnosis of hepatic damage, and also may aid to decrease the risk of adverse medication events and attaining the optimal drug concentration (17). COVID-19 cases with underlying liver damage are advised to be cured with medicines that could both prevent inflammatory responses and protect liver biochemistries, such as ammonium glycyrrhizinate that may, in turn, accelerate disease recovery (16). Basically, glycyrrhizin is a plant product isolated from licorice root. Glycyrrhizin is used for treating chronic hepatitis and is a relatively non-toxic product. A previous *in vitro* study demonstrated anti-SARS effects for glycyrrhizin by inhibiting viral attachment and entry, which was showed high effectivity when used both during and after the viral adsorption period (51). Although, the potential anti-COVID-19 effects of glycyrrhizin need further investigation. Docking findings were proposed that glycyrrhizin has the capability to bind ACE2 receptor (52). Regarding the lower toxicity of glycyrrhizin, its anti-viral properties on SARS and its potential to interact with ACE2, it is noteworthy to examine the efficacy of this herbal product against SARS-CoV-2 infection (52). Furthermore, Certain vitamins such as vitamin D and vitamin B12, magnesium and statins have been implicated for reducing the risk of COVID-19 infection or ameliorating severity of the disease (53). Also, zinc supplementation may play a potential role for prophylaxis and therapy of patients with COVID-19 infection (54).

## Important Considerations in COVID-19 Patients With Mild and Severe Liver Diseases

For COVID-19 cases with mild hepatic injury, the recommendations are similar to general population including perfect hand washing, social distancing, well-cough etiquette and avoiding sick people. The existence of liver disease may make diagnosis more complicated. COVID-19 elevates the liver enzymes and for patients who have more advanced liver disease, especially liver transplant waiting list candidates and post-transplant immunosuppression cases, the risk is higher. Immunosuppressed post-transplant recipients probably are at an elevated risk. These patients should not decide to stop immunosuppression treatment because of potential development of liver rejection. It deserves to be declared that immunosuppressed patients without fever may delay the diagnosis of COVID-19 infection. Routine laboratory checks should be postponed up to 3 weeks, but if common laboratory checks turn into a video visitation program, it will decrease the exposure of liver transplanted individuals. Also, social distancing is more important than physical distance (28).

## Conclusions

COVID-19 infection can involve multiple organs other than respiratory tract and lungs, in particular liver. A noticeable incidence of liver damage accompanied by abnormal ranges of AST, ALT, ALP, bilirubin, and albumin levels has been reported during COVID-19 disease. Different mechanisms have been suggested for liver injury during COVID-19 infection including immune-mediated hepatitis, direct CPE of the virus, drug-induced liver injury secondary to medications used for the treatment of COVID-19, infection-induced systemic inflammation, hepatic congestion secondary to PEEP ventilation and pre-existing liver disease. Hence, regular monitoring of liver functions in COVID-19 cases can result in an early diagnosis of liver disease. On the other hand, one of the major concerns during COVID-19 outbreak is the population with a history of pre-existing chronic liver diseases, their management, medical treatment, and important consideration. Liver injury cases are recommended to be treated with medications that could both prevent inflammatory responses and protect liver function. In addition, important considerations in COVID-19 patients with mild and severe liver diseases are highly advised. Further studies should focus on drugs application which may induce liver injury, such as antimicrobials (for example; macrolides) and the effect of liver-related comorbidities on treatment and consequence of COVID-19 infection.

## Author Contributions

SR contributed significantly to the literature review and wrote the first draft of the manuscript. BH worked on concept and design of the study and interpreted the collected clinical information. HA and MZ provided clinical advice and guidance for improving of the manuscript. AY, SR, and BH critically revised the final version of the manuscript. All authors approved the final version of the manuscript and the authorship list.

## Conflict of Interest

The authors declare that the research was conducted in the absence of any commercial or financial relationships that could be construed as a potential conflict of interest.
